# Highly mobile and reactive state of hydrogen in metal oxide semiconductors at room temperature

**DOI:** 10.1038/srep03149

**Published:** 2013-11-06

**Authors:** Wan Ping Chen, Ke Feng He, Yu Wang, Helen Lai Wah Chan, Zijie Yan

**Affiliations:** 1 Key Laboratory of Artificial Micro- and Nano-structures of Ministry of Education and School of Physics and Technology, Wuhan University, Wuhan 430072, China; 2 Department of Applied Physics and Materials Research Centre, The Hong Kong Polytechnic University, Hong Kong, China; 3 James Franck Institute, The University of Chicago, 929 East 57th Street, Chicago, Illinois 60637, United States

## Abstract

Hydrogen in metal oxides usually strongly associates with a neighboring oxygen ion through an O-H bond and thus displays a high stability. Here we report a novel state of hydrogen with unusually high mobility and reactivity in metal oxides at room temperature. We show that freshly doped hydrogen in Nb_2_O_5_ and WO_3_ polycrystals via electrochemical hydrogenation can reduce Cu^2+^ ions into Cu^0^ if the polycrystals are immersed in a CuSO_4_ solution, while this would not happen if the hydrogenated polycrystals have been placed in air for several hours before the immersion. Time-dependent studies of electrochemically hydrogenated rutile single crystals reveal two distinct states of hydrogen: one as protons covalently bonded to oxygen ions, while the other one is highly unstable with a lifetime of just a few hours. Observation of this mobile and reactive state of hydrogen will provide new insight into numerous moderate and low temperature interactions between metal oxides and hydrogen.

Hydrogen has been attracting extensive research interests aiming at exploring its versatile behaviors in crystalline solids^1^,^2^. Usually being introduced into metal oxides via annealing in H_2_ or H_2_O-containing atmospheres at elevated temperatures, many interesting, yet sometimes quite complicated features of hydrogen in metal oxides have been revealed^3^,^4^,^5^. They are meaningful for understanding hydrogen incorporated into metal oxides at high temperatures. However, metal oxide semiconductors usually interact with hydrogen at lower or even room temperature in their hydrogen-related applications, such as hydrogenation catalysis^6^, hydrogen storage^7^,^8^, and low-temperature hydrogen sensing^9^. It is well known that the interaction between metal oxides and hydrogen strongly depends on the reaction temperature. For instance, the reaction of MO_3_ (M = W or Re) with molecular hydrogen at 500°C leads to the formation of MO_2_, while that with dissociated hydrogen at room temperature forms hydrogen bronze H_x_MO_3_^10^. It is thus doubtful that the properties of hydrogen obtained from high-temperature hydrogen-doped metal oxides are adequate for understanding low-temperature hydrogen-related applications of metal oxide semiconductors. As a matter of fact, some investigations have indicated some very puzzling behaviors of hydrogen incorporated into metal oxides at moderate and low temperatures^8^,^11^,^12^, and some unusually unstable behaviors have also be observed for hydrogen in those low-temperature hydrogen-related applications of metal oxide semiconductors, yet so far our understanding of these behaviors is very limited^13^.

It is thus highly desirable to study metal oxides with hydrogen introduced at low, and if possible, room temperature. Electrochemical hydrogenation through electrolysis of water has been found to be a facile method to dope hydrogen at room temperature into a number of metal oxides, such as TiO_2_, Nb_2_O_5_, WO_3_, BaTiO_3_ and Pb(Zr,Ti)O_3_^14^,^15^,^16^,^17^,^18^. In this article, we will present some novel findings with regards to the behaviors of hydrogen in metal oxides obtained through this room temperature hydrogen doping method. In “freshly” hydrogenated Nb_2_O_5_ and WO_3_ polycrystals, a portion of the doped hydrogen apparently exists in a highly mobile and reactive state: a copper layer was deposited on the hydrogenated sample when it was immersed in CuSO_4_ solution immediately after hydrogenation, while the copper layer would not form if the hydrogenated sample had been aged for ten hours before the immersion. By electrochemically doping hydrogen into TiO_2_ (001) rutile single crystals, we found a wide step appeared at the low angle side of the (002) peak in X-ray diffraction (XRD); the step quickly narrowed and disappeared in a few hours while the distorted (002) peak (due to hydrogen insertion) would slowly recover over hundreds of hours, and the stretch mode of O-H bond was observed by infrared spectroscopy. These contrasts demonstrate two distinct states of hydrogen: one as protons covalently bonded to oxygen ions, while the other one being highly mobile and reactive, probably as isolated protons “free” to move in the oxide lattices.

## Results

**Figure 1:**
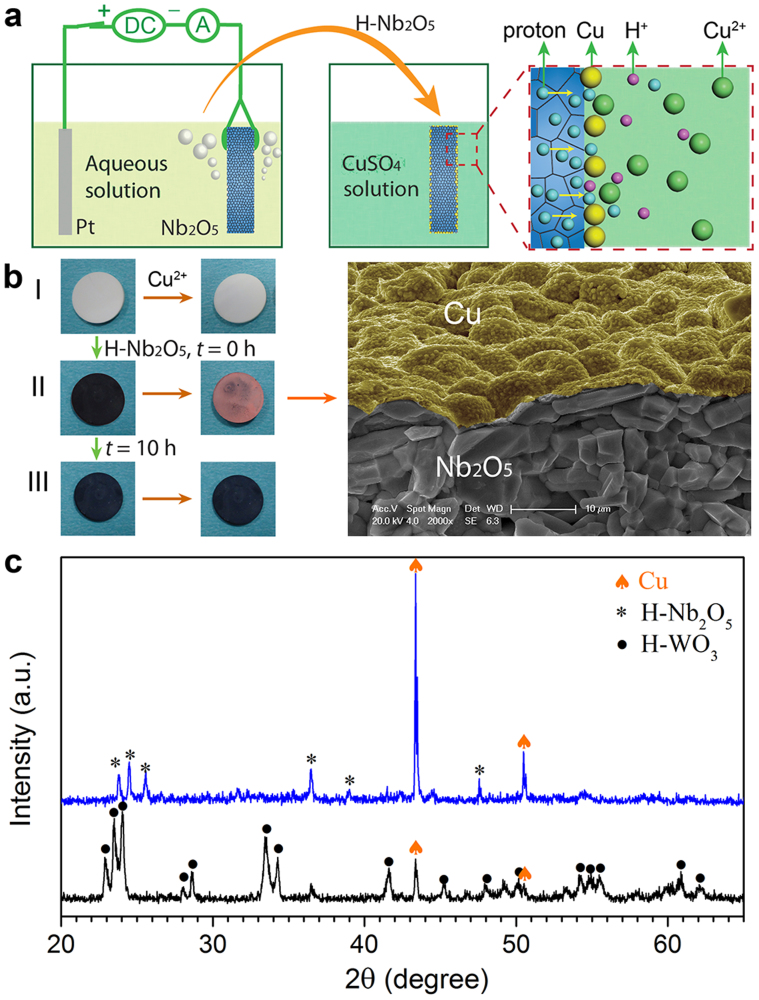
Highly reactive hydrogen in hydrogenated oxides. (a), Schematic of electrochemically hydrogenating an Nb_2_O_5_ ceramic pellet and then immersing it into a 0.5 M CuSO_4_ solution at room temperature. (b), Optical images of Nb_2_O_5_ pellets before and after being immersed in 0.5 M CuSO_4_ solution for 10 min: I. an as-sintered sample, II. a hydrogenated sample, III. a hydrogenated and 10 h-aged sample. The right side is a scanning electron microscopy image taken over the cross-section of an immersed sample. The Cu layer is shown with a false color. c, XRD pattern taken on the surface of the hydrogenated Nb_2_O_5_ pellet after 10 min immersion. A pattern taken on the surface of a hydrogenated WO_3_ pellet after 10 min immersion is also shown.

As illustrated in Fig. 1a, we electrochemically hydrogenated an Nb_2_O_5_ ceramic pellet for 24 h; the white color turned into deep blue due to the incorporation of hydrogen in the oxide lattices^15^. After that, we immediately immersed the pellet in a 0.5 M CuSO_4_ solution for 10 min at room temperature. Surprisingly, we observed a reddish layer appeared on the pellet surface, as shown in Fig. 1b. In contrast, if the hydrogenated pellet (another sample) was placed in air for 10 h at room temperature and then immersed in the CuSO_4_ solution, no such layer was deposited though it was still deep blue after 10 h of aging. XRD analysis reveals that the reddish layer is copper as shown in Fig. 1c, and the diffraction peaks of the Cu are much stronger than those of the Nb_2_O_5_ ceramics. Since Nb_2_O_5_ by itself could not reduce Cu^2+^ ions as shown in Fig. 1b, the only possible route of the formation of Cu is the reduction of Cu^2+^ by hydrogen from the hydrogenated Nb_2_O_5_. What should be emphasized is that in just 10 min the pellet surface was covered by a thick copper layer, indicating that a relatively large amount of doped hydrogen exists in a highly mobile and reactive state, which could diffuse out of the Nb_2_O_5_ and react with Cu^2+^ at the surface. The high mobility and reactivity also imply a short lifetime of such hydrogen, and after 10 h of aging they almost disappeared from the oxide lattices so no copper was deposited on the aged pellet during the immersion.

Nb_2_O_5_ is not the only oxide that can accommodate such highly mobile and reactive hydrogen. Indeed copper was also found to be deposited on a 24 h electrochemically hydrogenated WO_3_ pellet when it was immersed in 0.5 M CuSO_4_ solution for 10 min immediately after the hydrogenation, but the amount was much less than that formed on similarly hydrogenated Nb_2_O_5_ (see the XRD pattern in Fig. 1c). As a large amount of hydrogen can be electrochemically inserted into WO_3_, it is strange that only a little portion of inserted hydrogen seems to exist in the mobile and reactive state so the amount of deposited copper was very small. These results suggest that this special state of hydrogen may be not rare among metal oxides, while its concentration can be dramatically different among different metal oxides.

**Figure 2:**
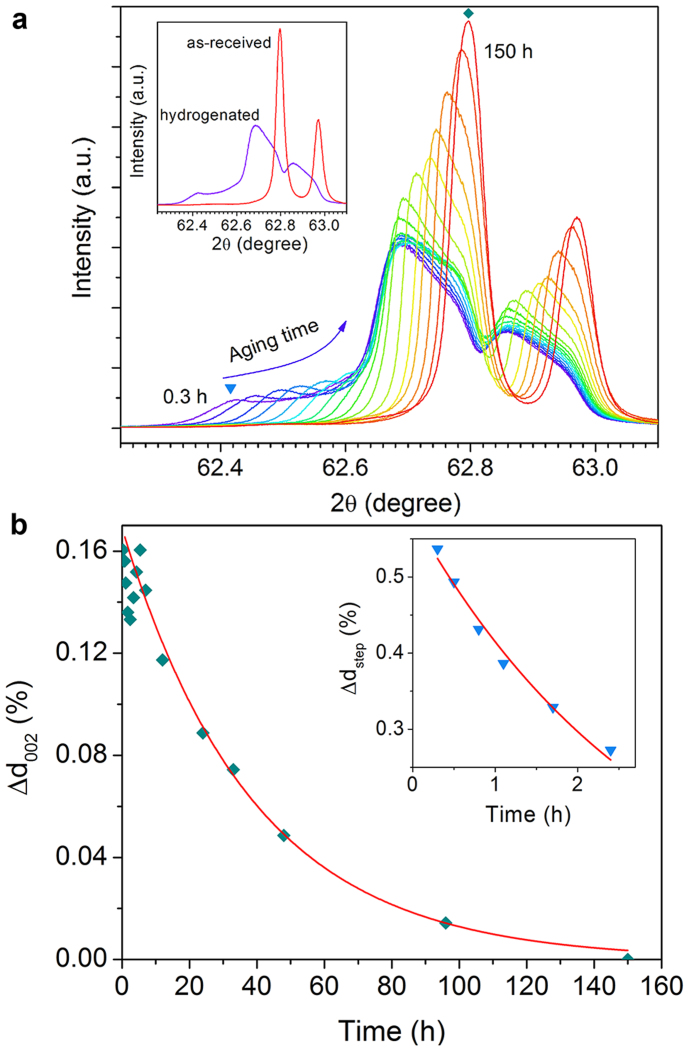
Crystal structure evolution of electrochemically hydrogenated TiO_2_. (a), XRD patterns of an electrochemically hydrogenated (001) rutile single crystal taken after a series of aging time. The hydrogenation time is 200 h, and the aging times are 0.3, 0.5, 0.8, 1.1, 1.7, 2.4, 3.4, 4.3, 5.4, 7, 12, 24, 33, 48, 96, and 150 h for the patterns from left to right. Inset: XRD patterns of an as-received (001) rutile single crystal and the electrochemically hydrogenated (001) rutile single crystal measured at an aging time of 0.3 h. (b), Lattice expansion of the (002) plane of the hydrogenated crystal compared to that of the undoped one as a function of aging time. The red curve is an exponential fit of the data: Δ*d* = 0.0017exp(–*t*/39). Inset: the lattice expansion of the wide step indicated by a triangle in panel a. The red curve is also an exponential fit: Δ*d* = 0.0058exp(–*t*/3).

We expect that it should be possible to reveal a more detailed evolution of this unstable hydrogen in metal oxide single crystals. As Nb_2_O_5_ and WO_3_ single crystals are not available, we turned to TiO_2_ rutile single crystals to conduct time-dependent study. These single crystals have nearly perfect crystal lattices that can eliminate the influence of crystal defects to doped hydrogen. For some metal oxides, phase transformation or crystal reformation occurs upon the hydrogen doping^16^. In this study, although we have tried to insert as much hydrogen as possible into the rutile single crystals, no new phases could be detected through XRD. Instead, the lattice of the rutile single crystals was found to be distorted to an extent beyond expectation when much hydrogen was inserted. Figure 2a shows XRD patterns of a hydrogenated rutile single crystal measured at different aging times, *t*, after 200 h of electrochemical hydrogen insertion. The (002) peak of as-received single crystals is sharp and highly symmetric (see the inset). In contrast, the (002) diffraction peak of the hydrogenated crystal becomes broad and is distorted to a very irregular shape, indicating non-uniform plane spacing or hydrogen distribution. An important observation is that there exists a wide step at the low angle side of the peak for short aging times, e.g., at 62.42° as indicated by the triangle for *t* = 0.3 h. We note that while the specific shape of the (002) peak could vary depending on the hydrogen insertion time, the broadening of the peak and the arising of a wide step always occur (see Supplementary Information, Fig. S1). Chester and Bradhurst have electrochemically inserted hydrogen into vacuum-reduced rutile single crystals and found that the hydrogen concentration gradient was much higher in the first few microns of the surface layer than in the bulk region (~100 μm deep)^19^. The wide step revealed in our study can be well explained in term of this layered structure: the concentration of hydrogen is particularly high at the surface, where the plane spacing is expanded by 0.54% at *t* = 0.3 h compared to that of the undoped (002) plane, while at the same time the expansion of the inner layer in the bulk region is only 0.16% as indicated by the distorted peak.

The XRD patterns further reveal that the distortion evolved with the aging time by placing the hydrogenated crystals in air at room temperature, indicating that the distorted crystal lattice as well as the layered structure subsequently experiences a spontaneous and systematic restoration as shown in Fig. 2b. The lattice expansion, Δ*d*, of the hydrogenated crystal decreases exponentially by 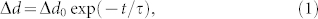
where Δ*d*_0_ is the initial lattice expansion and *τ* is a decay constant. For the case of the wide step, Δ*d*_0_ = 0.58% and *τ* = 3 h, indicating that the layered structure exists only for a few hours in our sample after the hydrogen insertion, and thus the hydrogen in the layer is highly mobile. In contrast, it takes longer aging time for the distorted peak to restore to the original state. The distorted (002) peak in Fig. 2a gradually decreases in width, and increases in height and symmetry following the eq. 1 with Δ*d*_0_ = 0.17% and *τ* = 39 h. After an aging time of *t* = 150 h, the peak has almost restored to its original highly sharp and symmetric shape. Spontaneous recovery was also observed for other initial distortions (Supplementary Information, Fig. S2). These two dramatically different decay constants exactly reflect the obvious difference in mobility observed for the step and the peak. The difference suggests that hydrogen in the surface layer is not only of a higher concentration but also much more mobile than that in the bulk region, so there exist two distinct states of hydrogen with different mobilities in these electrochemically hydrogenated rutile single crystals. This is consistent with the observations that only the “freshly” hydrogenated Nb_2_O_5_ and WO_3_ polycrystals could reduce Cu^2+^ ions but those aged samples could not. In other words, highly unstable hydrogen has been observed in all these three metal oxides that were investigated in this study, although its concentration is dramatically different in different metal oxides.

**Figure 3:**
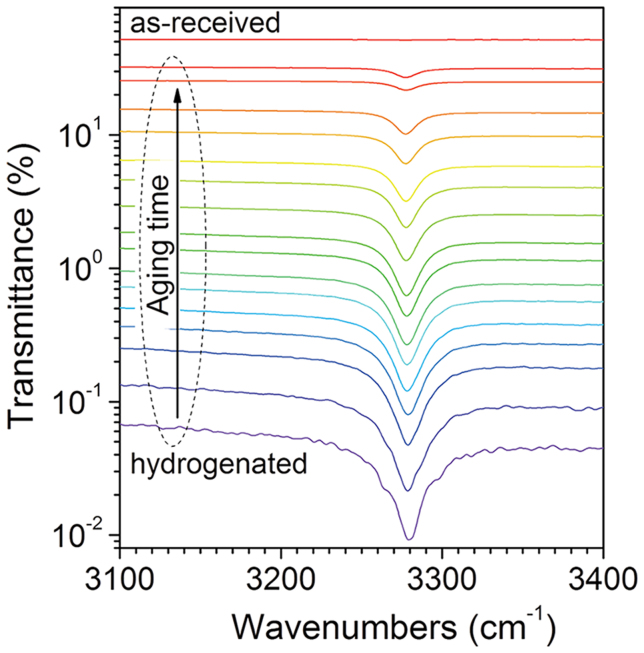
Infrared absorption spectra of an as-received rutile single crystal and a hydrogenated one measured after a series of aging times. The hydrogenation time is 120 h, and the aging times are 2, 9, 24, 34, 48, 72, 144, 192, 310, 500, 750, 1150, 1550, 2400, and 3000 h for the spectra from bottom to top.

Figure 3 shows infrared absorption spectra measured at 300 K for a rutile single crystal that has been electrochemically hydrogenated for 120 h and an as-received rutile single crystal. The as-received rutile single crystals are colorless and almost transparent without clear absorption peak in the range of 3100–3400 cm^−1^ that is common for as-grown rutile single crystals. In contrast, the hydrogenated sample was deep-blue in color and its transparency was greatly decreased. A strong and broad absorption peak centered at 3279 cm^−1^ can be clearly observed. This peak is continuously weakened with increasing aging time and meanwhile the transmittance of the hydrogen-inserted rutile single crystal is increased. It is well known that the infrared absorption peak at 3279 cm^−1^ is due to the stretch mode of O-H bond at room temperature in rutile. So this peak can be assigned to the stretch mode of O-H bond^20^. As a matter of fact, electrochemically doped hydrogen has been found to greatly increase the conductivity and act as a shallow donor in rutile single crystals^21^. No other hydrogen-related structures can be observed over a wide frequency range of 500–4000 cm^−1^ (Supplementary Information, Fig. S3), and it is necessary to point out that hydrogen can exist in metal oxides in some forms that are not seen by IR spectroscopy^22^,^23^. Obviously, for the two distinct states of hydrogen revealed by XRD, the one with a bigger decay constant can be identified as protons covalently bound to oxygen ions according to the infrared absorption spectra analysis, which can be expressed as 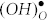
24; while the highly mobile one is still largely unknown, e.g., its charge state that can be either positive, neutral or negative^25^. For simplicity, we tentatively assume a positive charge state for it and term it as 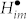 (*i* for interstitial and *m* for mobile) hereinafter.

## Discussion

Hydrogen existing as 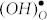 is well-known for metal oxides and is found in many applications of metal oxides. For example, for electrochromic metal oxide WO_3_, 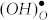 is charged/discharged reversibly through controlling applied voltage^10^, which can be expressed using the notation of Kröger and Vink in the following way: 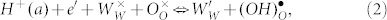
where 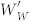 represents a W^5+^ at the lattice site of W^6+^ in WO_3_ and *H*^+^(*a*) a proton in a solution. The electrochromic effect is directly related the formation of 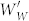, namely W^5+^ in the lattice. It should be pointed out that the formation of O-H bond tends to weaken the ferroelectric distortion in WO_3_^10^ and with increasing content of 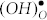, the crystal structure is modified from monoclinic (WO_3_) to tetragonal (H_0.1_WO_3_ and H_0.33_WO_3_), and to cubic (H_0.5_WO_3_)^16^. Through thermal treatment, it leaves from the lattice of WO_3_ as water^26^: 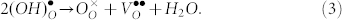
In contrast, as a different state, hydrogen existing as 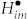 is expected to leave the oxide lattice in a different form rather than H_2_O. A mass spectroscopy analysis of the gas released from electrochemically hydrogenated Nb_2_O_5_ upon thermal treatment included a large portion of H_2_ besides H_2_O^8^,^26^. Considering that H_2_O is formed from 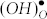, H_2_ should be formed from 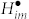, which exists in electrochemically hydrogenated Nb_2_O_5_, upon thermal treatment in the following way: 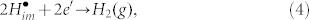
where the electron is weakly-bonded or free in the metal oxide and the out-diffusion of hydrogen decreases the concentration of charge carriers and thus the conductivity of metal oxides. As for the deposition of copper on “freshly” hydrogenated Nb_2_O_5_ and WO_3_ pellets when being immersed in the CuSO_4_ solution, it reveals the following reaction between 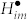 and Cu^2+^ at room temperature: 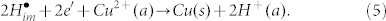
As 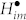 can be of a high concentration in hydrogenated Nb_2_O_5_, a large portion of H_2_ is formed besides H_2_O upon thermal treatment, and a large amount of copper is formed when immersed in CuSO_4_ solution at room temperature. On the other hand, since the content of 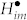 is very small in electrochemically hydrogenated WO_3_, it is difficult to collect H_2_ upon heat-treatment and only H_2_O can be detected, and the amount of copper deposition is very small when immersed in CuSO_4_ solution.

It is clear that with such surprisingly high mobility and reactivity at room temperature, this special state of hydrogen will not be observed or studied in samples treated through hydrogen-doping at elevated temperatures. Our finding of the highly mobile and reactive state of hydrogen, and this room-temperature hydrogen doping method as well, are thus especially valuable for studying those interactions between metal oxides and hydrogen at moderate and low temperatures. For example, the peculiar aging behavior of electrochemically hydrogenated TiO_2_ rutile single crystals^14^,^21^, and some other metal oxides as well^12^,^18^,^27^, can now be better understood: the mobile hydrogen 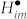 should play an important role in the initial stage of aging, while 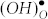 should be mainly responsible for the subsequent aging^28^. For hydrogen-sensing of metal oxide semiconductors, it is generally considered that the change of electrical resistance with hydrogen exposure is only related to the spilt hydrogen atoms on the surface^9^,^29^,^30^, in this way electron transfer from hydrogen to metal oxides occurs while hydrogen is still highly mobile. According to our present results, however, ionized hydrogen, or proton, can actually exist inside metal oxides while being highly mobile. So it is necessary to examine whether hydrogen will enter into the lattice of metal oxides in the course of hydrogen-sensing. Similarly, hydrogen entrance into the lattice of metal oxides should also be considered for other chemisorption of atomic hydrogen on the surface of metal oxides^31^,^32^. Nb_2_O_5_ is so far the most effective oxide promoter for accelerating adsorption/desorption of hydrogen in magnesium hydride storage materials. It has been assumed that Nb_2_O_5_ may play the role as a reservoir loading a certain amount of split hydrogen in its lattice in the course of catalysis^8^,^11^,^26^. Obviously, our results provide a direct support for this assumption and more attention should be paid to it when the catalysis mechanism of Nb_2_O_5_ is studied.

In summary, by studying electrochemically hydrogenated Nb_2_O_5_ and WO_3_ polycrystals, and TiO_2_ rutile (001) single crystals, we found the coexistence of hydrogen in two distinct states inside the lattice of these metal oxides: one is as protons attached to oxygen ions by strong O-H bonds while the other one is highly mobile and reactive, but exists only for several hours in the lattice. The properties of this mobile and reactive hydrogen are still largely unknown, and further investigations need to be conducted on this special state of hydrogen, such as what its actual charge state is, how 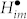 interacts with metal oxide lattices, how 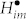 interacts with other crystal defects like 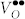, and how, if possible, 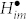 and 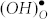 are transformed into each other. Our results are therefore important for deepening our understanding of hydrogen in metal oxides and for promoting numerous hydrogen-related applications of metal oxides.

## Methods

Nb_2_O_5_ samples were prepared through 2 h sintering at 1250°C in air of pellets (15 mm in diameter and 2 mm thick) pressed from analytical grade (99%) Nb_2_O_5_ powders. Analytical grade (99%) WO_3_ powder was first ball milled with de-ionized water for 24 h. After drying, the milled powder was pressed into pellets of 15 mm in diameter and 2 mm thick and the pellets were sintered at 1150°C for 2 h in air. The TiO_2_ rutile single crystals used in this work were (001)-oriented 10 × 10 × 0.5 mm^3^ commercial wafers purchased from HEFEI KEJING (China). Two 2 mm diameter silver electrodes were fired on the two sides of a corner of the single crystals. Electrochemical hydrogen insertion was conducted by immersing a sample in a 0.01 M NaOH solution. The Nb_2_O_5_ or the WO_3_ pellets or the silver electrodes of the rutile single crystals were connected to dc voltages (6 V) to act as the cathode to electrolyze water. Infrared absorption spectra were recorded with a Nicolet iS-10 Fourier transform spectrometer by measuring the transmittance of as-received and hydrogenated rutile single crystals at 300 K. The spectral resolution was 0.5 cm^−1^. X-ray diffraction patterns were taken through an X-ray diffractometer (Philips PW 3719) with Cu Kα radiation.

## Author Contributions

W.P.C. designed the research and conducted the experiments. W.P.C. and Z.Y. wrote the manuscript. K.F.H. contributed to the measurements. Z.Y., Y.W. and H.L.W.C. contributed to the project design and data analysis. All authors discussed the results and commented on the paper.

## Supplementary Material

Supplementary Information SUPPLEMENTARY INFO

## References

[b1] Van de Walle C. G. Hydrogen as a cause of doping in zinc oxide. Phys Rev Lett 85, 1012–1015 (2000). 10991462 10.1103/PhysRevLett.85.1012

[b2] Kilic C. & Zunger A. n-type doping of oxides by hydrogen. Appl Phys Lett 81, 73–75 (2002).

[b3] Shi G. A. *et al.* Hydrogen local modes and shallow donors in ZnO. Phys Rev B 72, 195211 (2005).

[b4] Spahr E. J. *et al.* Giant of hydrogen transport in rutile TiO_2_ at low temperatures. Phys Rev Lett 104, 205901 (2010). 20867041 10.1103/PhysRevLett.104.205901

[b5] Tarun M. C. & McCluskey M. D. Infrared absorption of hydrogen-related defects in strontium titanate. J Appl Phys 109, 063706 (2011).

[b6] Marcq J. P., Poncelet G. & Fripiat J. J. Hydrogenation by hydrogen bronzes: II. Hydrogenation of ethylene by H_x_V_2_O_5_. J Catal 87, 339–351 (1984).

[b7] Berzins A. R. & Sermon P. A. Reversible isothermal sorption of hydrogen by tungsten trioxide in presence of platinum. Nature 303, 506–508 (1983).

[b8] Dolci F., Di Chio M., Baricco M. & Giamello E. Niobium pentoxide as promoter in the mixed MgH_2_/Nb_2_O_5_ system for hydrogen storage: a multitechnique investigation of the H_2_ uptake. J Mater Sci 42, 7180–7185 (2007).

[b9] Varghese O. K. *et al.* Extreme changes in the electrical resistance of titania nanotubes with hydrogen exposure. Adv Mater 15, 624–627 (2003).

[b10] Dickens P. G., Crouchbaker S. & Weller M. T. Hydrogen insertion in oxides. Solid State Ionics 18–9, 89–97 (1986).

[b11] Borgschulte A., Rector J. H., Dam B., Griessen R. & Zuttel A. The role of niobium oxide as a surface catalyst for hydrogen absorption. J Catal 235, 353–358 (2005).

[b12] Yao W. Q. *et al.* Hydrogen-induced degradation and aging of Pb(Mg_1/3_Nb_2/3_)O_3_-based X7R multilayer ceramic capacitors. Jpn J Appl Phys 47, 5530 (2008).

[b13] Kreuer K.-D. Proton conductivity: materials and applications. Chem Mater 8, 610–641 (1996).

[b14] Chen W. P. *et al.* Spontaneous recovery of hydrogen-degraded TiO_2_ ceramic capacitors. Appl Phys Lett 84, 103–105 (2004).

[b15] Chen W. P. *et al.* Hydrogen as an unstable shallow donor in oxides. Jpn J Appl Phy 49, 051103 (2010).

[b16] Chen W. P. *et al.* Effects of electrochemical hydrogen charging on electrical properties of WO_3_ ceramics. J Mater Sci 42, 2524–2527 (2007).

[b17] Chen W. P., Jiang X. P., Wang Y., Peng Z. & Chan H. L. W. Water-induced degradation of barium titanate ceramics studied by electrochemical hydrogen charging. J Am Ceram Soc 86, 735–737 (2003).

[b18] Shafiei A., Nickchi T., Oprea C., Alfantazi A. & Troczynski T. Investigation of hydrogen effects on the properties of Pb(Zr,Ti)O_3_ in tetragonal phase using water electrolysis technique. Appl Phys Lett 99, 212903 (2011).

[b19] Chester P. F. & Bradhurst D. H. Electrolytically induced conductivity in rutile. Nature 199, 1056 (1963).

[b20] Johnson O. W., Paek S. H. & DeFord J. W. Diffusion of H and D in TiO_2_: suppression of internal fields by isotope exchange. J Appl Phys 46, 1026 (1975).

[b21] Chen W. P., Wang Y. & Chan H. L. W. Hydrogen: a metastable donor in TiO_2_ single crystals. Appl Phys Lett 92, 112907 (2008).

[b22] Fillaux F., Ouboumour H., Tomkinson J. & Yu L. T. An inelastic neutron scattering study of the proton dynamics in γ-MnO_2_. Chem Phys 149, 459–469 (1991).

[b23] Shi G. A., Saboktakin M., Stavola M. & Pearton S. J. “Hidden hydrogen” in as-grown ZnO. Appl Phys Lett 85, 5601–5603 (2004).

[b24] Iwahara H. in: Colomban P. (ed).Proton Conductors: Solids, Membranes, and Gels: Materials and Devices. Cambridge University Press, New York, 1992.

[b25] Kobayashi Y. *et al.* An oxyhydride of BaTiO_3_ exhibiting hydride exchange and electronic conductivity. Nature Mater 11, 507–511 (2012). 22504535 10.1038/nmat3302

[b26] Dolci F., Di Chio M., Baricco M. & Giamello E. The interaction of hydrogen with oxidic promoters of hydrogen storage in magnesium hydride. Mater Res Bull 44, 194–197 (2009).

[b27] Chen W. P., Wang Y., Chan H. L. W. & Luo H. S. Hydrogen-related dynamic dielectric behavior of barium titanate single crystals. Appl Phys Lett 88, 202906 (2006).

[b28] Herklotz F., Lavrov E. V. & Weber J. Infrared absorption of the hydrogen donor in rutile TiO_2_. Phys Rev B 83, 235202 (2011).

[b29] Lu C., Chen Z. & Yu P. A novel reaction model for the electrical conductivity of ultra-thin TiO_2_ films in H_2_. Phys Chem Chem Phys 13, 9131–9133 (2011). 21503315 10.1039/c1cp20180h

[b30] Wang Z. *et al.* Fast and highly-sensitive hydrogen sensing of Nb_2_O_5_ nanowires at room temperature. Intl J Hydrogen Energy 37, 4526–4532 (2012).

[b31] Roland U., Braunschweig T. & Roessner F. On the nature of spilt-over hydrogen. J Mol Catal A: Chem 127, 61–84 (1997).

[b32] Roland U., Salzer R., Braunschweig T., Roessner F. & Winkler H. Investigations on hydrogen spillover: Part 1.–electrical conductivity studies on titanium dioxide. J Chem Soc-Faraday Trans 91, 1091–1095 (1995).

