# Supplementary material for: Highly mobile and reactive state of hydrogen in metal oxide semiconductors at room temperature
Source: Sci Rep. 2013 Nov 6;3:3149. doi: 10.1038/srep03149 (PMC3818659; doi:10.1038/srep03149)
Supplement: Supplementary Information — SUPPLEMENTARY INFO [file srep03149-s1.doc]

**Supplementary Information**

**Highly mobile and reactive state of hydrogen in metal oxide semiconductors at room temperature**

Wan Ping Chen^1,*^ Ke Feng He,^1^ Yu Wang,^2^ Helen Lai Wah Chan,^2^ and Zijie Yan^3,*^

^1^School of Physics and Technology, Wuhan University, Wuhan 430072, China. ^2^Department of Applied Physics and Materials Research Centre, The Hong Kong Polytechnic University, Hong Kong, China. ^3^James Franck Institute, The University of Chicago, 929 East 57th Street, Chicago, Illinois 60637, United States.


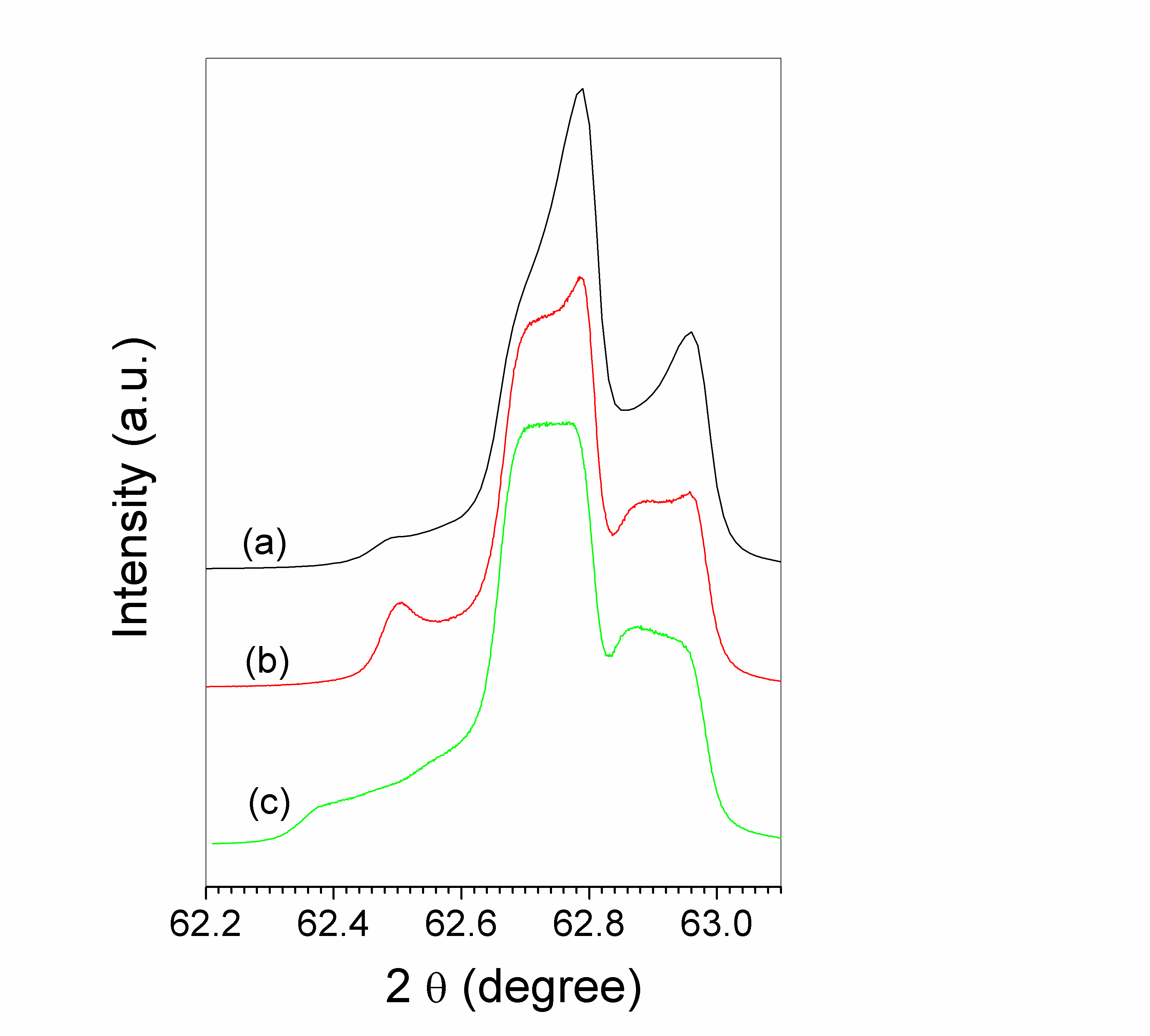


**Figure S1.** X-ray diffraction patterns (a) a rutile single crystal measured at 0.3 h after 50 h of hydrogen insertion; (b) a rutile single crystal measured at 1 h after 100 h of hydrogen insertion; and (c) a rutile single crystal measured at 0.3 h after 150 h of hydrogen insertion.


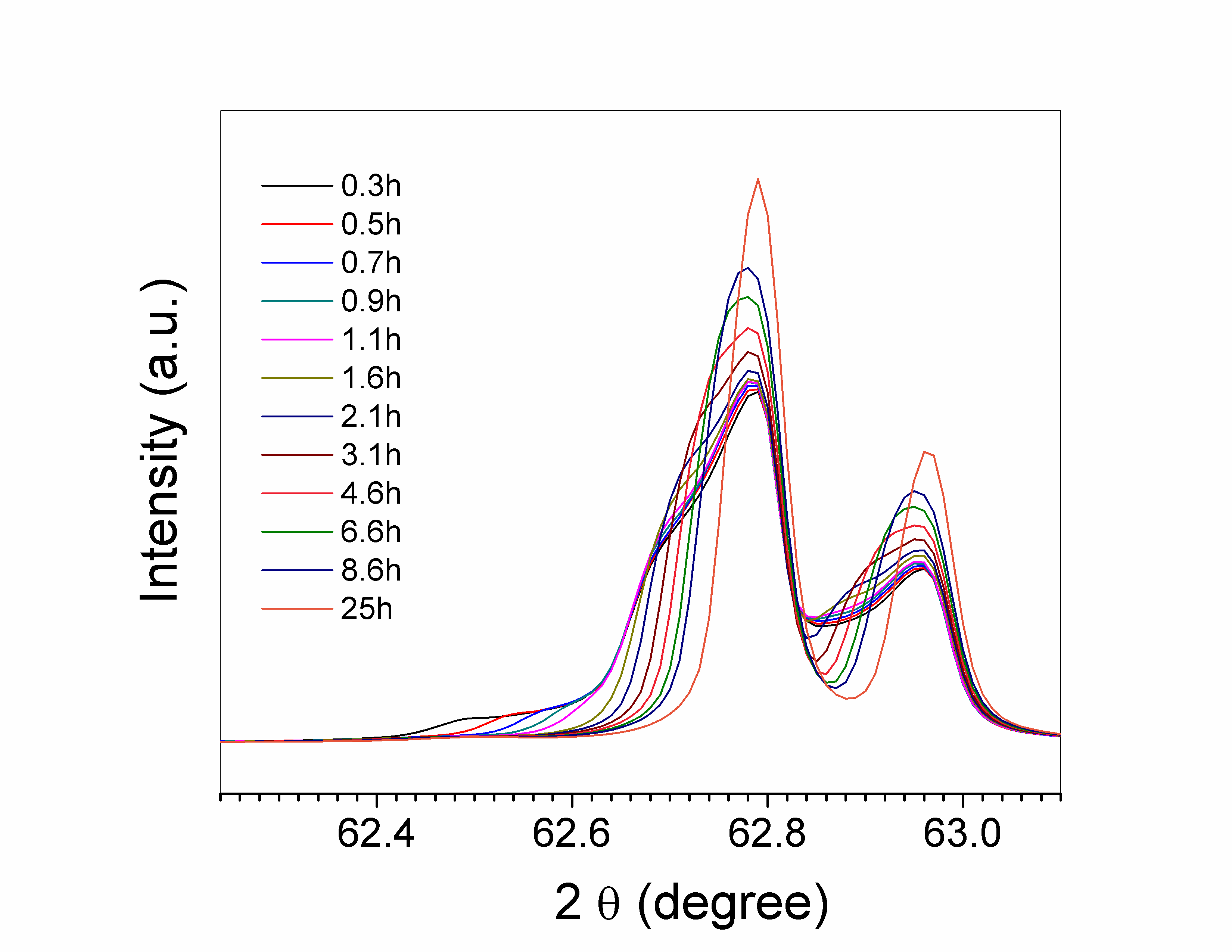


**Figure S2.** Structure evolution of hydrogenated rutile single crystal. X-ray diffraction patterns of the 50 h hydrogenated rutile single crystal measured after a series of aging time.


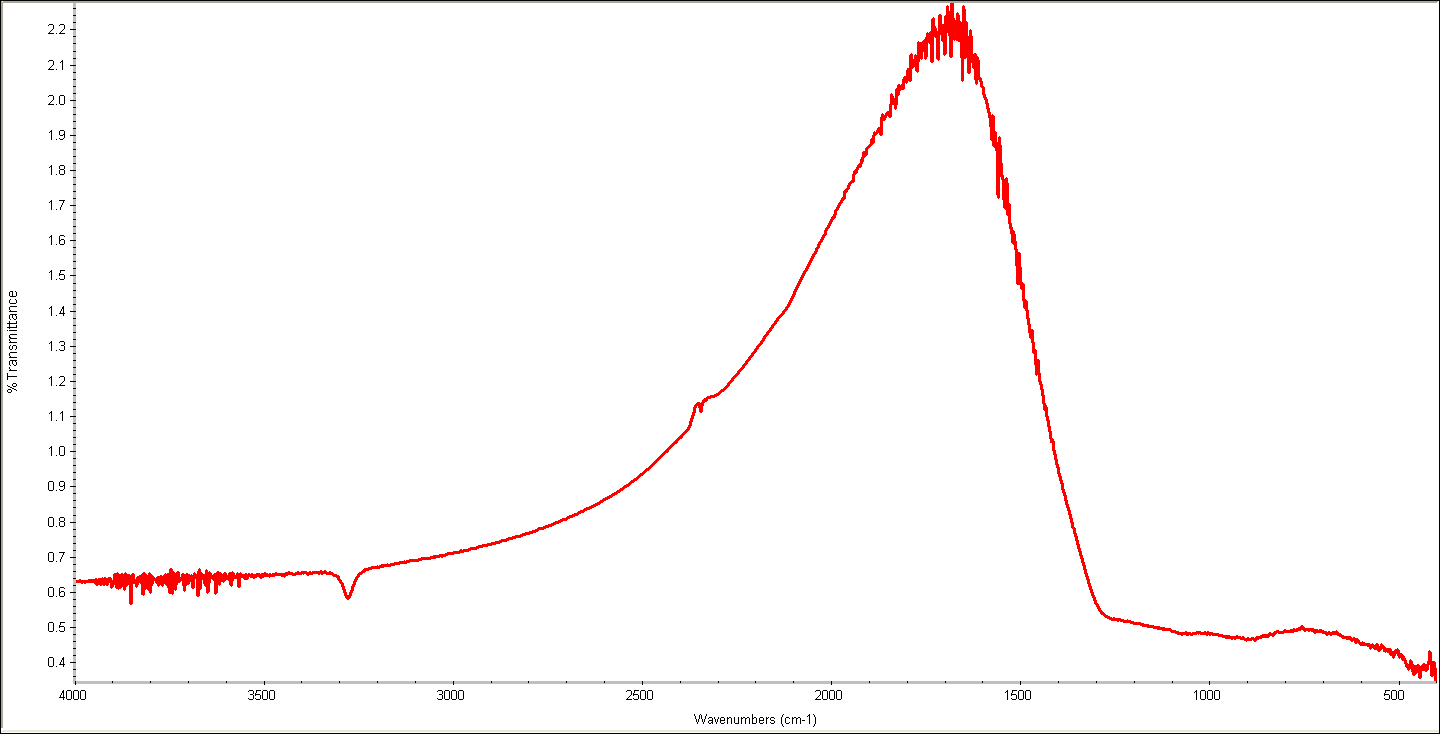


**Figure S3.** Infrared absorption spectrum of a hydrogenated rutile single crystal measured after 24 h of aging. The hydrogenation time is 200 h.
